# Treatment for Hæmoptysis

**Published:** 1905-04-22

**Authors:** 


### TREATMENT FOR HÆMOPTYSIS.

Serious hæmoptysis in the course of tuberculosis of the lungs is so alarming both to the patient and the physician, that any treatment of real value is assured of a hearty welcome. Dr. Francis Hare,[1] dealing with the matter, discusses the theoretical possibilities which should guide treatment. These are:

1. Constriction of the branches of the pulmonary artery. This has been attempted by the use of adrenalin, but without success. It has, we fancy, been experimentally established that the pulmonary circulation is entirely uninfluenced by this drug, except insomuch as the general blood-pressure is raised thereby. It appears, therefore, that the exhibition of adrenalin is actually contra-indicated.

2. Reduction in the force of the heart beats. This is the object of the accepted treatment by rest and the avoidance of stimulants. Aconite has been used for a like purpose, but Dr. Hare has had no experience of it in this connection.

3. Reduction of the blood-pressure. This would seem to be the most rational indication, for it is nature's spontaneous means for the arrest of hæmorrhage. For this purpose Dr. Hare recommends amyl nitrite in the highest terms. He has tried it in 17 attacks occurring in nine subjects. In 15 of the total the cessation of serious bleeding was instantaneous. In one case the bleeding ceased in three minutes, and in the remaining one, in ten minutes. In five cases only did recurrence take place, and then only after some hours. In all cases the recurrent hæmorrhage yielded readily to the drug.

This method is a modern application of an old principle. Dr. Hare quotes the ancient practice of bleeding for hæmoptysis, and there is another antiquated and heroic method which we have seen successfully employed as a desperate resource in a very bad case—viz. the administration of a vomiting dose of ipecacuanha. In all these the same object is attained, though in the one case by peripheral vaso-dilation, in the second by actual depletion, and in the third by cardiac depression. Dr. Hare's method certainly seems a great advance on its predecessors.

[1] Clin. Jour., March 22, 1905.

